# Association of Kidney Function Tests with a Cardio-Ankle Vascular Index in Community-Dwelling Individuals with a Normal or Mildly Decreased Estimated Glomerular Filtration Rate

**DOI:** 10.3390/medicina55100657

**Published:** 2019-09-29

**Authors:** Javad Alizargar, Chyi-Huey Bai, Nan-Chen Hsieh, Shu-Fang Vivienne Wu, Shih-Yen Weng, Jia-Ping Wu

**Affiliations:** 1 Research Center For Healthcare Industry Innovation, National Taipei University of Nursing and Health Sciences, Taipei City 112, Taiwan; 2 Department of Public Health, College of Medicine, Taipei Medical University, Taipei11031, Taiwan; 3 Department of Information Management, National Taipei University of Nursing and Health Sciences, Taipei City 112, Taiwan; 4 College of Nursing, School of Nursing, National Taipei University of Nursing and Health Sciences, Taipei City 112, Taiwan

**Keywords:** atherosclerosis, cardiovascular disease, cardio-ankle vascular index, ankle brachial index, hypertension, atherosclerosis, glomerular filtration rate

## Abstract

*Background and objectives*: Chronic kidney disease (CKD) is an independent risk factor for cardiovascular disease (CVD). Previous studies reported controversial results about the independence of CKD as a risk factor for atherosclerosis. In this study, we tried to determine whether the estimated glomerular filtration rate (eGFR) and other renal function tests are independent factors associated with arterial stiffness in community-dwelling individuals with a normal (≥90) or slightly decreased eGFR (60-90). *Materials and Methods*: Data of 164 community individuals were analyzed, and demographic information, related disease history, atherosclerosis risk factors, certain laboratory tests, the estimated eGFR, and urine albumin creatinine ratio (UACR) were recorded for each individual. *Results*: The age, systolic blood pressure (SBP), hypertension (HTN), and cardio-ankle vascular index (CAVI) significantly differed between individuals with a normal and those with a slightly decreased eGFR. Blood urea nitrogen (BUN), glycated hemoglobin (HBA1c), and the eGFR significantly differed between the high- and low-CAVI groups and were also significantly correlated with the CAVI. The relationship between the eGFR and CAVI was shown to be independent of other atherosclerosis risk factors in a multiple linear regression model. *Conclusions*: We concluded that evaluations of the eGFR, HTN, body-mass index, and SBP can be used in a model for arterial stiffness risk assessments for community-dwelling individuals with a normal or slightly decreased eGFR.

## 1. Introduction

Chronic kidney disease (CKD) is an independent risk factor for cardiovascular disease (CVD) and atherosclerosis [1]. Atherosclerosis is more prevalent in CKD patients, and it also causes more comorbidities in CKD patients [2]. Studies have produced controversial results as to the independence of CKD indexes as risk factors for atherosclerosis among other atherosclerosis risk factors. Some studies stated that atherosclerosis is associated with early-stage CKD [3], while other studies stated that this association is not independent of traditional risk factors of atherosclerosis [4].

Arterial stiffness can be estimated by different indexes like the carotid intima medial thickness (cIMT), the ankle brachial index (ABI), and carotid-femoral pulse wave velocity (cfPWV). The cfPWV used to be the most frequently used index for assessing atherosclerosis. The cfPWV and other indexes mentioned above are all dependent on changes in blood pressure (BP) [5]. The cardio-ankle vascular index (CAVI) is a new index that was introduced to resolve this issue [6]. The CAVI is considered to be one of the best known indexes for atherosclerotic CVD risk prediction, as it is independent of BP changes. Studies suggest that CAVI serves as a better index compared to other indexes such as ABI for the evaluation of subclinical atherosclerosis [7].

The estimated glomerular filtration rate (eGFR), calculated by an equation of the CKD-epidemiology collaboration (EPI) using serum creatinine (Cr), age, sex, and race in the formula, was introduced and has become an easy method to evaluate renal function. The eGFR and urine albumin to creatinine ratio (UACR) are commonly used in evaluating CKD patients [8]. Some studies show the relationship of atherosclerosis (as measured by ABI) and the GFR decline in the individuals [9], but studies on the relationship of CAVI and GFR are lacking.

The UACR and eGFR were used as renal function tests in this study. The main objective of this study was to evaluate the correlation of renal function tests with CAVI in community-dwelling individuals with a normal (≥90) or slightly decreased (60-89) eGFR. The independence of the association of renal function tests with CAVI was also evaluated by considering classical atherosclerosis risk factors that might also influence CAVI measurements. We also try to depict a comparison between the relationship of CAVI and ABI in correlation with eGFR.

## 2. Materials and Methods

All of the participants in this study were part of a large community-based prospective cohort that evaluated CVD risk factors. The study field was the Shihlin and Wenshan Districts of Taipei, Taiwan. During the original study, participants were recruited every year from 2005 for CVD evaluations. After approval of the Ethics Committee of Taipei Medical University in conjunction with the Institutional Review Board (IRB) (reference nos. 94E-183, 94E-198, and 96E-004), the CAVI and renal function tests were added to the study for the 2017 recruitment. Details of the original study and inclusion and exclusion criteria of the study participants were published elsewhere [10]. As a summary, all of the participants in the study fields were recruited by telephone and invited to visit the hospital. Exclusion criteria were being aged less than 30 years, having an incomplete questionnaire, with a prior history of cancer or CKD, and refusing to have blood drawn or to allow the CAVI measurement. To ensure that our study had a sufficient sample size, after collecting data of participants, the power for comparing the two means for evaluating the CAVI between the two eGFR groups (with a two-sided 95% confidence interval (CI)) was calculated, and if it was not >80%, then more participants were recruited to reach 80% power.

In total, 198 individuals visited the hospital in 2017 to participate in this study. All participants gave their informed consent for inclusion before the original study and also in every year in which they responded. The study was conducted in accordance with the Declaration of Helsinki. All individuals’ demographic data and CAVI measurements were recorded. The age, sex, body height and weight, a personal history of ischemic heart disease (IHD) and type 2 diabetes mellitus (T2DM), and a family history of heart disease (FH) and hypertension (HTN) were recorded. The presence of IHD and T2DM was based on a physician’s diagnosis. An FH was positive if there was a history of heart disease in a brother or father less than 60 years old or in a sister or mother before 65 years of age. The smoking history was also determined as whether they had ever smoked or not, and if so, the number of packs of cigarettes per day was multiplied by the duration of smoking in years to calculate the pack-years of smoking.

The habit of alcohol consumption and the volume (in mL) of alcoholic drinks (5% alcohol) per week were investigated. Participants were asked whether they had the habit of exercising and the frequency of exercising per week. Fasting blood was taken and sent to the laboratory for determination of serum levels of blood urea nitrogen (BUN), Cr, aspartate aminotransferase (AST), alanine aminotransferase (ALT), fasting blood sugar (FBS), serum uric acid (UA), serum triglyceride (TGL), cholesterol (CHOL), low-density lipoprotein (LDL), high-density lipoprotein (HDL), high-sensitivity C-reactive protein (Hs-CRP), glycated hemoglobin (HBA1c), and urine albumin and creatinine (for calculating their ratio) using standard methods (with X-1500-Sysmex (Norderstedt, Germany), Beckman AU5800 (Brea, CA, USA), and Tosoh HLC-723G8 automated glycol-hemoglobin analyzer (Tokyo, Japan)).

For the CAVI measurement, participants were asked to lie down in a supine position for 10 min, and an electrocardiogram (ECG) and phonocardiogram (PCG) were performed. The pulse wave velocity (PWV) was measured by VaSera VS-1000 (Fukuda Denshi, Tokyo, Japan) All CAVI and ankle/brachial index (ABI) measurements were done intrinsically with the VS-1000. The CAVI was determined by the following formula: CAVI = a [(2 ρ /ΔP) × ln (SBP/DBP) PWV^2^] + b; where SBP and DBP are the systolic and diastolic BPs, ΔP is (SBP – DBP), and ρ is the blood density. a and b are constants automatically measured by the device to match the aortic PWV. High and low CAVI values were assessed based on the sex and age group of the individuals as referenced to a study by Namekata et al. [11].

The eGFR was calculated using the CKD-EPI equation [12]: eGFR = 141 × min (S_cr_/κ, 1)^α^ × max (S_cr_/κ, 1)^-1.209^ × 0.993^Age^ × 1.018 [if female] × 1.159 [if black]; where S_cr_ is serum Cr in mg/dL, κ is 0.7 for females and 0.9 for males, α is −0.329 for females and −0.411 for males, min indicates the minimum of S_cr_/κ or 1, and max indicates the maximum of S_cr_/κ or 1. The GFR is considered stage 1 (normal) if ≥90 and stage 2 (mildly decreased) if 90 > GFR > 60. Proteinuria was assessed as the UACR.

Among the 198 individuals who participated in our study, nine individuals with a prior history of CVD admission, one with stroke admission, two with hepatitis C infection, 14 with hepatitis B infection, two with an ABI of >1.4, and eight with a GFR of <60 were also excluded. That left data of 164 individuals for use in the final analysis. All study variables were analyzed by an analysis of variance (ANOVA) and Fisher’s exact test stratified by CAVI and GFR levels. The correlation of the eGFR with CAVI was analyzed using Pearson’s correlation test. A multiple linear regression test was used to analyze associated risk factors of atherosclerotic CVD and the eGFR. All statistical analyses were performed using SAS 9.4 (SAS Institute, Cary, NC, USA).

## 3. Results

Data of 164 community-dwelling individuals were analyzed. The mean age ± standard deviation (SD) was 62.64 ± 9.47. Sixty-two participants (37.80%) were male. The mean eGFR ± SD was 91.96 ± 10.94 mL/min/1.73 m^2^. Table 1 summarizes study parameters stratified by GFR levels. Secondary analysis by keeping all the 198 individuals showed that analysis of baseline parameters base on GFR in Table 1 were not affected by the exclusion of those 34 individuals and the excluded individuals had the same baseline characteristics (Table S1).

We evaluated levels of laboratory test results based on the CAVI group. To evaluate correlations of laboratory tests with the CAVI, a Pearson’s correlation test was run. Results are presented in Table 2.

Analysis on the relationship of lipid profile and eGFR shows that there is not a significant correlation between LDL, HDL, and TGL with eGFR as the Pearson’s correlation coefficients between LDL, HDL, and TGL with eGFR are −0.003, 0.038, and −0.022 (all *p* values > 0.05).

**Figure 1:**
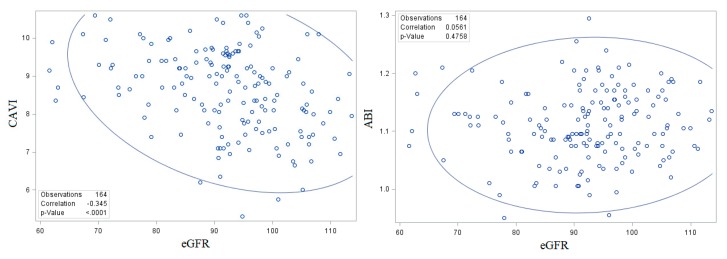
Scatter plot with 95% prediction ellipse between CAVI and eGFR (**left**) and between ABI and eGFR (**right**). eGFR, estimated glomerular filtration rate (mL/min/1.73 m^2^); CAVI, cardio-ankle vascular index; ABI, ankle brachial index.

In order to observe the relationship between CAVI and eGFR, a correlation analysis was done and the scatter plot to show the relationship between these two parameters was depicted. The correlation coefficient was −0.345 and the *p* value < 0.001. The plot can be seen in Figure 1.

We performed a multivariate regression analysis to evaluate the independence of the association of the eGFR with the CAVI (*R*^2^ = 0.331). Results of this analysis are shown in Table 3.

## 4. Discussion

This study was performed on 164 community-dwelling individuals, and results suggested that age, SBP, HTN, and CAVI differed between individuals with a normal eGFR and those with a slightly decreased eGFR. BUN, HBA1c, and the eGFR significantly differed between levels of the CAVI and were significantly correlated with the CAVI. The relationship between the eGFR and CAVI was shown to be independent of other atherosclerotic CVD risk factors. Previous studies showed the relationship between other arterial stiffness indexes and the eGFR. Wu et al. [3] reported that the eGFR was correlated with the cIMT according to Pearson’s correlations of −0.346 for males and −0.253 for females. Parv et al. [13] showed that the GFR was independently associated with the cIMT, ABI, carotid plaque, femoral IMT and plaque, and flow-mediated dilation in postmenopausal women. Although our results are consistent with those studies (we found a Pearson’s correlation of −0.344), our measure of atherosclerosis was the CAVI, and our study subjects were community-dwelling individuals (and included both men and women).

Some studies pointed out the effects of T2DM and CKD on atherosclerosis [13]. Although we found that the eGFR, a marker of CKD, was independently and significantly correlated with atherosclerosis, T2DM was not found to be correlated with atherosclerosis. This might have been because of the relatively small sample size in our study. Studies with higher numbers of T2DM patients are needed to confirm this relationship. We also found no difference between the ABI in patients with different levels of the eGFR, but other studies, for example Parv et al.’s study [13], found a relationship between the ABI and eGFR. Although our study subjects and atherosclerosis indexes differed between our study and theirs, this might also have been due to the exclusion of abnormal ABI individuals in our study. More studies are needed to confirm the relationship of ABI with atherosclerosis.

Maebuchi et al. [14] found that the CAVI can be used as a predictive marker for the incidence of CKD in non-CKD patients. Although their study showed the value of the CAVI for CKD prediction, a CKD parameter (eGFR) was found to be associated with the CAVI as a marker of atherosclerosis. The association of the eGFR measurement as an independent factor for atherosclerosis was shown in a study by Chen et al. [15]. Several pathologic factors were proposed to explain the value of the eGFR in arterial stiffness, including secondary hyperparathyroidism, elevated homocysteine levels, lipoprotein (a) metabolism, imbalances between calcium and phosphate, alterations in inflammatory and coagulation pathways, fluid overload, alterations in the angiotensin and endothelin systems, malnutrition, elevated uremic toxins, oxidative stress, and insulin resistance [16,17].

Another factor that should be considered in the renal injury is the diet. Although protein restriction in the diet of individuals with existing kidney disease can be beneficial, there is not much evidence that this restriction can help the individuals without kidney disease to avoid renal injury [18]. On the other hand, high fat diet consumption has an important effect on renal disease. This role might be due to the renal lipid accumulation and the increases in inflammatory cytokines. High fat consumption diet induces glomeruli retraction and renal dysfunction [19]. Unfortunately, the data on the diet of the individuals were not available in our study and we suggest that future studies contain information about the participants’ high fat consumption diet and consider it as an important covariate in their analysis. About the role of lipid profile on GFR, some studies show that triglyceride level is independently associated with GFR [20]. We found no association between lipid profile and eGFR in our study and confirming the presence or lack of this relationship needs more studies on this field.

Ours is the first study to evaluate the independence of the association of renal function tests with the CAVI. As low eGFR was independently associated with risk of cardiovascular events [21], and we found that CAVI is associated with eGFR, therefore studies that can link CAVI to cardiovascular events due to the impact on renal function can link CAVI to cardiovascular events in individuals with renal function impairment. Some studies have been working on ABI as the atherosclerosis index [10] and showed the relationship of these markers but as we found out the relationship between CAVI and eGFR seems to be more robust as shown in the univariate and multivariate regression analysis. Our study power for comparing the two means, for evaluating the CAVI between the two eGFR groups was 98.39% (two-sided 95% CI) [22]. Our study also had several limitations. It only compared stages 1 and 2 in CKD, and higher stages with lower eGFRs (<60%) could not be evaluated. We also excluded individuals with an abnormal ABI, and certain CVDs like stroke. Therefore, our study results are only generalizable to community-dwelling individuals without those diseases. Although our study was mainly based on a reliable arterial stiffness index (CAVI), including other stiffness indexes like the baPWV would have also been beneficial for enabling us to compare our study results to other studies. Therefore, we suggest that future studies include different subjects and other indexes of arterial stiffness. Furthermore, we could not include data on pharmacotherapy in this study due to massive missing data regarding the use of medications in our participants. We strongly suggest considering the use of medications such as antihypertensives, statins, diuretics, etc., which could impact on glomerular filtration rate and make an impact on the measured endpoints in the future studies. Another shortcoming in this study is that the participants were recruited from a large community-based cohort and the number of individuals included in this study is small. Therefore, the inclusion bias can threat the validity of the results and must be considered in the future studies. At last, although eGFR was independently related to CAVI in a statistical model this does not necessarily mean that eGFR independently modifies the atherosclerotic process. Presence of such conclusions should be confirmed by prospective cohorts.

## 5. Conclusions

We concluded that the eGFR evaluation in community-dwelling individuals with a normal or slightly decreased eGFR can be significantly and independently associated with arterial stiffness. Evaluations of the eGFR, HTN, BMI, and SBP can be used in a model for arterial stiffness risk assessment. Measurement of the eGFR as an independent factor can be added to current models to predict atherosclerosis for screening purposes in the first stages of CKD.

## Figures and Tables

**Table 1:** Distribution of study parameters at different levels of the glomerular filtration rate (GFR).

Parameter	Mean ± SD Number (%)	GFR (mL/min/1.73 m^2^)		
		60-89	≥90	*p* value
Overall	164 (100)	56 (34.15)	108 (65.85)	**-**
Age (years)	62.64 ± 9.47	69.64 ± 7.40	59.01 ± 8.34	**<0.001**
Sex (male)	62 (37.80)	26 (46.43)	36 (33.33)	0.126
BMI (kg/m^2^)	24.80 ± 3.61	24.87 ± 3.30	24.76 ± 3.78	0.853
WC (cm)	80.50 ± 9.89	81.61 ± 10.07	79.92 ± 9.79	0.301
HC (cm)	94.04 ± 6.87	93.58 ± 6.39	94.28 ± 7.13	0.537
ABI	2.22 ± 0.12	2.19 ± 0.11	2.23 ± 0.12	0.128
SBP (mmHg)	130.04 ± 17.44	134.17 ± 16.30	127.89 ± 17.69	**0.028**
DBP (mmHg)	80.66 ± 10.36	81.57 ± 10.40	80.19 ± 10.36	0.421
T2DM	23 (14.02)	10 (17.86)	13 (12.04)	0.346
HTN	55 (33.54)	26 (46.43)	29 (26.85)	**0.014**
IHD	12 (7.32)	7 (12.5)	5 (4.63)	0.109
FH	37 (22.84)	17 (30.36)	20 (18.87)	0.116
SMK	31 (18.90)	11 (19.64)	20 (18.52)	0.837
Pack-years	0.57 ± 3.70	0.45 ± 3.40	0.63 ± 3.86	0.770
Alc	57 (34.76)	18 (32.14)	39 (36.11)	0.729
Alc. vol. (mL)	106.52 ± 252.84	90.53 ± 266.80	114.81 ± 246.16	0.561
Exercise	131 (79.88)	45 (80.36)	86 (79.63)	1.00
Exe. freq. per week	3.24 ± 3.23	3.69 ± 3.20	3 ± 3.24	0.198
CAVI	8.62 ± 1.08	9.04 ± 0.83	8.40 ± 1.14	**<0.001**

BMI, body-mass index; WC, waist circumference; HC, hip circumference; ABI, ankle brachial index; SBP, systolic blood pressure; DBP, diastolic blood pressure; T2DM, type 2 diabetes mellitus; HTN, hypertension; IHD, ischemic heart disease; FH, a family history of heart disease; SMK, with a smoking habit; Alc, with a habit of alcohol consumption; 5% Alc. vol., volume of alcoholic drinks (5%) consumed in a week; Exercise, with a habit of exercising; Exe. freq. per week, number of times exercising per week; CAVI, cardio-ankle vascular index; SD, standard deviation; GFR, glomerular filtration rate.

**Table 2:** Distribution of study laboratory results stratified by cardio-ankle vascular index (CAVI) levels and their correlations with the CAVI.

Test	Mean ± SD Number (%)	CAVI			Pearson Correlation with the CAVI	
		Low	High	*p* value	*r*	*p* value
BUN (mg/dL)	14.58 ± 3.45	14.38 ± 3.41	15.19 ± 3.55	**0.193**	**0.207**	**0.007**
Cr (mg/dL)	0.741 ± 0.155	0.74 ± 0.15	0.73 ± 0.14	0.643	−0.003	0.961
AST (units/L)	19.01 ± 4.57	19.02 ± 5	19 ± 2.98	0.976	0.132	0.090
ALT (units/L)	18.10 ± 7.78	18.21 ± 8.35	17.75 ± 5.80	0.742	0.034	0.660
FBS (mg/dL)	93.43 ± 22.12	91.71 ± 21.05	98.58 ± 24.63	0.085	0.124	0.111
UA (mg/dL)	5.33 ± 1.21	5.25 ± 1.25	5.59 ± 1.09	0.123	0.077	0.321
TGL (mg/dL)	117.38 ± 52.59	115.74 ± 55.07	122.29 ± 44.58	0.491	0.059	0.452
CHOL (mg/dL)	190.64 ± 30.78	191.20 ± 31.11	188.97 ± 30.06	0.689	0.013	0.861
LDL (mg/dL)	115.99 ± 28.04	115.33 ± 28.61	117.97 ± 26.50	0.602	0.028	0.715
HDL (mg/dL)	53.74 ± 12.34	54.05 ± 12.71	52.80 ± 11.24	0.575	0.014	0.855
hs-CRP (mg/L)	0.183 ± 0.34	0.160 ± 0.193	0.25 ± 0.60	0.148	0.122	0.119
HBA1c (%)	5.93 ± 0.80	5.85 ± 0.71	6.18 ± 0.98	**0.024**	**0.234**	**0.002**
eGFR (mL/min/1.73 m^2^)	91.96 ± 10.94	93 ± 11.02	88.82 ± 10.20	**0.033**	**–0.344**	**<0.001**
UACR (mg/g)	12.58 ± 25.21	13.38 ± 28.09	10.2113.49	0.510	0.066	0.420

BUN, blood urea nitrogen; Cr, serum creatinine; AST, aspartate aminotransferase; ALT, alanine aminotransferase; FBS, fasting blood sugar; UA, serum uric acid; TGL, serum triglyceride; CHOL, cholesterol; LDL, low-density lipoprotein; HDL, high-density lipoprotein; Hs-CRP, high-sensitivity C-reactive protein; HBA1c, glycated hemoglobin; eGFR, estimated glomerular filtration rate; UACR, urine albumin creatinine ratio.

**Table 3:** Multivariate linear regression* for evaluation of factors associated with the cardio-ankle vascular index (CAVI).

Variable	Unstandardized Beta Coefficients	Standardized Beta Coefficients	*p* Value
BMI	−0.006	−0.176	**0.025**
eGFR	−0.002	−0.185	**0.029**
HTN	0.068	0.247	**0.003**
SBP	0.001	0.179	**0.021**

* adjusted for Alc., with an alcohol consumption habit; Alc. vol., volume of alcohol consumed in a week; T2DM, type 2 diabetes mellitus; Exercise, with an exercise habit; SMK, with a smoking habit; LDL, low-density lipoprotein; HDL, high-density lipoprotein; Hs-CRP, high-sensitivity C-reactive protein; BUN, blood urea nitrogen; TGL, serum triglyceride; UA, serum uric acid. Abbreviations used in the table: BMI, body-mass index; eGFR, estimated glomerular filtration rate (mL/min/1.73 m^2^); HTN, hypertension; SBP, systolic blood pressure.

## Supplementary Materials

The following are available online at https://www.mdpi.com/1010-660X/55/10/657/s1. Table S1. Distribution of study parameters at different levels of the glomerular filtration rate (GFR) in all the individuals before the exclusion of 34 individuals from the study.
