# Supplementary material for: Association of Kidney Function Tests with a Cardio-Ankle Vascular Index in Community-Dwelling Individuals with a Normal or Mildly Decreased Estimated Glomerular Filtration Rate
Source: Medicina (Kaunas). 2019 Sep 29;55(10):657. doi: 10.3390/medicina55100657 (PMC6843344; doi:10.3390/medicina55100657)
Supplement: Supplementary file 1 [file medicina-55-00657-s001.pdf]

Table S1. Distribution of study parameters at different levels of the glomerular filtration rate (GFR) in all the individuals before the exclusion of 34 individuals from the study

Parameter	Mean±SD<br>Number (%)	GFR (ml/min/1.73 m^2)		
		60~90	≥90	*p* value
Overall	198 (100)	74(37.37)	124(62.63)	-
Age (years)	63.42±9.37	70.21±7.15	59.37±8.12	**<0.001**
Sex (male)	83 (41.92)	38 (51.35)	36(36.29)	0.052
BMI (kg/m^2)	24.78±3.61	24.61±3.26	24.89±3.81	0.604
WC (cm)	80.68±9.97	81.34±9.61	80.29±10.19	0.477
HC (cm)	94.04±6.87	92.99±6.20	94.45±7.18	0.147
ABI	2.21±0.12	2.19±0.12	2.23±0.11	0.078
SBP (mmHg)	130.02±17.31	134.12±15.87	127.57±17.73	**0.009**
DBP (mmHg)	80.72±10.37	81.51±10.43	80.25±10.35	0.408
T2DM	28 (14.14)	14 (18.92)	14 (11.29)	0.145
HTN	70 (35.35)	37 (50.00)	33 (26.61)	**0.001**
IHD	22 (11.11)	12 (16.22)	10 (8.06)	0.101
FH	49 (24.74)	21 (28.37)	28 (22.58)	0.181
SMK	45 (22.73)	20 (27.03)	25 (20.16)	0.295
Pack-years	0.89±4.68	1.31±6.00	0.64±3.68	0.333
Alc	73 (36.87)	28 (37.84)	45 (36.29)	0.879
Alc. vol. (ml)	129.89±308.40	153.78±384.54	115.64±253.03	0.401
Exercise	161 (81.31)	62 (83.78)	99 (79.84)	0.573
Exe. freq. per week	3.47±3.42	3.97±3.53	3.18±3.34	0.116
CAVI	8.64±1.11	9.06±0.96	8.38±1.11	**<0.001**

BMI, body-mass index; WC, waist circumference; HC, hip circumference; ABI, ankle brachial index; SBP, systolic blood pressure; DBP, diastolic blood pressure; T2DM, type 2 diabetes mellitus; HTN, hypertension; IHD, ischemic heart disease; FH, a family history of heart disease; SMK, with a smoking habit; Alc, with a habit of alcohol consumption; 5 % Alc. vol., volume of alcoholic drinks (5%) consumed in a week; Exercise, with a habit of exercising; Exe. freq. per week, number of times exercising per week; CAVI, cardio-ankle vascular index; SD, standard deviation; GFR, glomerular filtration rate.
